# Anterior Perineal Hernia following Robot-Assisted Radical Cystectomy in a Male Patient: A Case of Transperineal Mesh Repair

**DOI:** 10.70352/scrj.cr.26-0310

**Published:** 2026-06-20

**Authors:** Yusuke Sato, Takashi Kinoshita, Koji Komori, Akira Ouchi, Hironobu Yasuoka, Takahiro Kojima, Tetsuya Abe

**Affiliations:** 1 Department of Gastroenterological Surgery, Aichi Cancer Center Hospital, Nagoya, Aichi, Japan; 2 Department of Urology, Aichi Cancer Center Hospital, Nagoya, Aichi, Japan

**Keywords:** perineal hernia, cystectomy, mesh repair, pelvic floor, urethrectomy, transperineal approach

## Abstract

**INTRODUCTION:**

Perineal hernia following radical cystectomy is exceedingly rare, and to our knowledge, no individual case report of anterior perineal hernia after robot-assisted radical cystectomy (RARC) with urethrectomy in a male patient has been described in the English-language literature. We report such a case, which was successfully repaired via a transperineal approach using mesh reinforcement.

**CASE PRESENTATION:**

An 80-year-old man was incidentally diagnosed with an anterior perineal hernia containing small bowel on routine postoperative follow-up CT, 4 years after RARC, urethrectomy, and ileal conduit diversion for muscle-invasive bladder cancer with prostatic invasion. The patient was initially asymptomatic and managed conservatively; however, he subsequently developed symptoms consistent with subacute small bowel obstruction, for which surgical repair was indicated. Surgery was performed via a transperineal approach. After resection of the hernia sac and reduction of the small bowel, the hernia defect (2.5 × 2.5 cm) was repaired using a 4-armed (cruciate) mesh anchored anteriorly and laterally to the posterior surface of the pubic bone and the bilateral inferior pubic rami, and posteriorly to a cord-like fibrous structure representing perineal remnants, enabling tension-free reconstruction without injury to the rectum. No recurrence was observed at 6 months postoperatively.

**CONCLUSIONS:**

This case highlights that perineal hernia can occur as a rare but clinically significant complication following radical cystectomy, even in male patients. A transperineal approach with mesh reinforcement, using the pubic bone and perineal remnants as fixation points, may represent a viable reconstructive option. Surgeons should be aware of this complication and select an individualized approach based on anatomical findings and surgical history.

## Abbreviations

APR
: abdominoperineal resection
RARC
: robot-assisted radical cystectomy

## INTRODUCTION

Perineal hernia is a rare type of hernia occurring in the perineal region and is classified as either primary, caused by weakness of the pelvic floor musculature, or secondary, developing after pelvic surgery.^1)^ Perineal hernia following APR is well recognized; however, its occurrence after radical cystectomy is exceedingly rare.^2)^ Although a limited number of cases have been reported in female patients,^3,4)^ to our knowledge, no individual English-language case report has described anterior perineal hernia after RARC with urethrectomy in a male patient. Herein, we report such a rare case and describe the anatomical rationale for successful transperineal mesh repair in the post-cystectomy male pelvis.

## CASE PRESENTATION

**Fig. 1:**
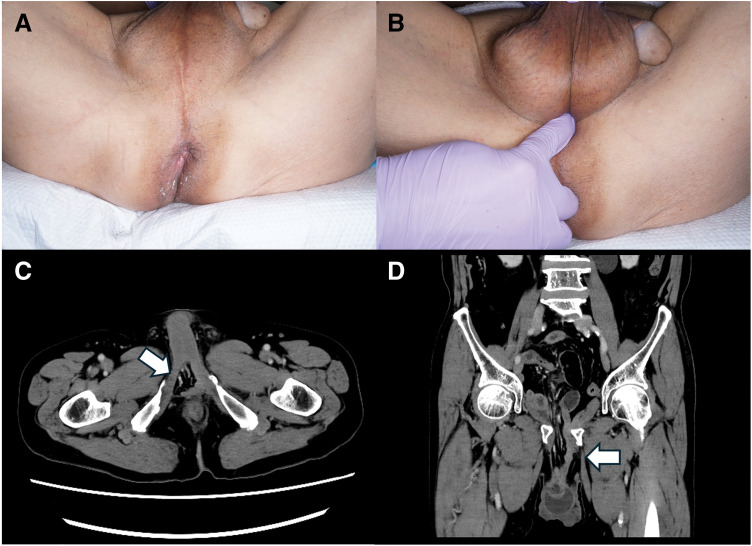
Clinical and radiological findings of the perineal hernia. (**A**) Supine position. No perineal protrusion is observed as the hernia is spontaneously reduced. (**B**) Digital palpation. A finger is easily admitted into the hernia orifice, confirming a distinct pelvic floor defect. (**C**, **D**) CT. Axial (**C**) and coronal (**D**) scans reveal the small bowel herniating anterior to the rectum and sliding into the scrotum.

An 80-year-old man was referred to our department after a routine postoperative follow-up CT scan incidentally revealed a perineal hernia containing small bowel. Four years earlier, he had been diagnosed with muscle-invasive bladder cancer with prostatic invasion. He received 2 cycles of neoadjuvant chemotherapy with gemcitabine and cisplatin, followed by RARC, urethrectomy, and ileal conduit diversion. CT imaging demonstrated an anterior perineal hernia, with small bowel protruding through a pelvic floor defect anterior to the rectum toward the scrotum (**Fig. 1C** and **1D**).

At the time of initial evaluation, the patient was asymptomatic. In the supine position, no perineal protrusion was visible as the hernia was spontaneously reduced (**Fig. 1A**). On physical examination, digital palpation confirmed a distinct pelvic floor defect, with a finger easily admitted into the hernia orifice (**Fig. 1B**). The bulge was more prominent in the standing position, without signs of incarceration. Given the absence of symptoms, conservative management was initially adopted. However, he subsequently developed intermittent lower abdominal discomfort, abdominal distension, and symptoms consistent with subacute small bowel obstruction, for which surgical repair was indicated.

**Fig. 2:**
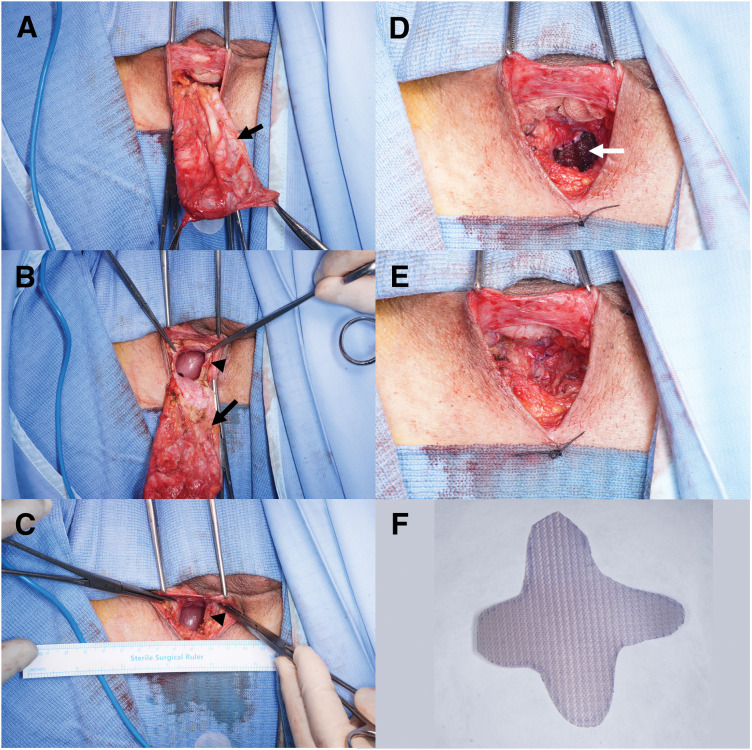
Intraoperative findings during transperineal mesh repair. (**A**) Dissection of the hernia sac (arrow) from the scrotum. The sac had extended into the scrotum and was carefully mobilized from the surrounding tissues, including the scrotal fascia. (**B**) Resection of the hernia sac (arrow) along the margin of the defect after complete dissection, with the hernia orifice (arrowhead) exposed. (**C**) The hernia orifice (arrowhead) measured approximately 2.5 cm in diameter. (**D**) Operative view after mesh fixation. The mesh (open arrow) is secured along the rim of the hernia orifice. Gauze packing is visible in the scrotal dead space. (**E**) Operative view after closure of the subcutaneous fascia. (**F**) Four-armed (cruciate) mesh configuration, tailored to provide approximately 3 cm of overlap beyond the hernia defect margin in each direction.

Surgery was performed under general anesthesia with the patient in the lithotomy position using a perineal approach. An incision was made along the previous surgical scar. The hernia sac was identified and carefully dissected from the surrounding tissues, including the scrotal fascia (**Fig. 2A**). After full exposure of the hernia orifice, the sac was resected along the margin of the defect (**Fig. 2B**). The small bowel showed no adhesions and was easily reduced into the abdominal cavity.

The hernia defect measured 2.5 cm intraoperatively (**Fig. 2C**). A Ventralight mesh (Bard Davol, Warwick, RI, USA) was tailored into a 4-armed (cruciate) configuration (**Fig. 2F**), which was chosen to ensure adequate overlap for pelvic floor reinforcement while minimizing wrinkling and folding of the mesh in areas where the peritoneum was absent and the mesh would be in direct contact with the bowel. Each arm was designed to provide approximately 3 cm of overlap beyond the hernia defect margin. The mesh was secured using 2 interrupted 3-0 nonabsorbable monofilament sutures at each fixation point: anteriorly to the periosteum of the posterior surface of the pubic bone, laterally to the bilateral inferior pubic rami, and posteriorly to a cord-like fibrous structure located posterior to the hernia orifice, presumed to represent remnants of the transverse perineal muscle, perineal body, and perineal membrane (**Fig. 2D**). In addition, approximately 10 interrupted sutures were placed circumferentially along the rim of the hernia orifice to reinforce fixation to the surrounding tissues, resulting in a total of approximately 18 sutures. This provided stable, tension-free fixation while maintaining adequate distance from the rectum. The subcutaneous fascia was then closed (**Fig. 2E**).

**Fig. 3:**
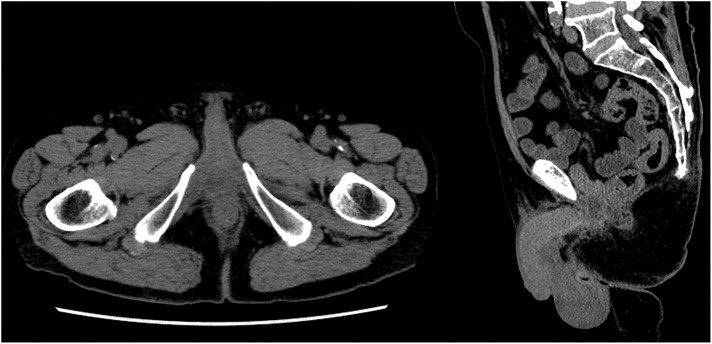
CT at 6 months postoperatively showing no evidence of hernia recurrence.

The postoperative course was uneventful, and the patient was discharged without complications. At 6 months postoperatively, CT showed no evidence of hernia recurrence (**Fig. 3**), and no mesh-related complications were observed. We acknowledge that 6 months represents a short-term follow-up only, and longer observation will be required to fully evaluate the durability of the repair.

## DISCUSSION

Perineal hernia is a well-recognized complication following APR for rectal cancer, with a reported incidence of 0.6%–13% after APR and potentially higher rates following minimally invasive procedures, likely owing to reduced postoperative adhesion formation.^1,5,6)^ In contrast, perineal hernia following radical cystectomy is exceedingly rare. The published literature consists predominantly of individual case reports in female patients.^3,4,7)^ Alvarez Garzón et al. reported an anterior perineal hernia after robotic radical cystectomy in a woman and performed transperineal mesh repair,^3)^ and Wong et al. described a similar complication following anterior exenteration.^4)^ To our knowledge, the present case is the first individually reported case of anterior perineal hernia after RARC with urethrectomy in a male patient. It adds a new perspective by demonstrating that the anatomical structures specific to the post-cystectomy pelvis—including bony landmarks anteriorly and laterally and residual perineal fibromuscular tissue posteriorly—can serve as reliable fixation points for tension-free mesh repair.

The rarity of perineal hernia in male patients after radical cystectomy may be explained in part by sex-related differences in pelvic floor anatomy. In males, the urogenital triangle is largely occupied by the bulbospongiosus, ischiocavernosus, and superficial transverse perineal muscles, which may provide greater structural resistance to herniation.^8)^ The male levator ani also forms a steeper and narrower funnel compared with the wider female pelvis, potentially limiting the space available for herniation.^9)^ However, these anatomical advantages are likely only 1 component of a multifactorial picture. In female patients, the vagina represents a potential site of structural weakness in the anterior pelvic floor, and differences in pelvic morphology and the extent of anterior resection may further contribute to sex-related differences in hernia risk. In the present case, RARC with urethrectomy disrupted the urogenital diaphragm and created an anterior pelvic floor defect of sufficient size to permit herniation, rendering the male pelvic floor vulnerable despite its anatomical advantages. Advanced age and the minimally invasive nature of the robotic procedure, which is associated with reduced adhesion formation and therefore less natural support of the pelvic floor, may also have contributed.

Both transabdominal and transperineal approaches have been described for perineal hernia repair. The transabdominal approach provides a wide operative field and has been associated with lower recurrence rates,^1)^ but carries greater invasiveness and the risk of intra-abdominal complications. The transperineal approach offers direct access to the hernia orifice with lower morbidity, and mesh reinforcement via this route has been shown to achieve outcomes comparable to the transabdominal approach.^10,11)^ In the present case, the hernia was small and fully reducible, and direct access to the hernia orifice was preferable. A transperineal approach was specifically chosen to avoid the risk of injury to the ileal conduit and intra-abdominal small bowel. Because the anus was preserved—unlike in APR—careful attention was paid to contamination control, including perioperative prophylactic antibiotics and intraoperative coverage of the anal region. During dissection, particular attention was paid to avoiding injury to the rectum posteriorly and to the ileal conduit and small bowel deep to the hernia orifice. The depth of posterior fixation was guided by the palpable cord-like fibrous structure rather than by blind deep dissection, which allowed safe placement of sutures without compromising the rectal wall.

A fundamental principle of hernia repair is secure mesh fixation to anatomically stable structures. In perineal hernia following APR, mesh fixation is commonly achieved using the residual levator ani, ischial tuberosity, coccygeus muscle, and coccyx.^10,12)^ The anatomical configuration in the present case, however, differed substantially from that seen after APR. The absence of the prostate and urethra following RARC left an anterior pelvic floor defect whose anterior and lateral borders were defined by bony structures—the posterior surface of the pubic bone and the bilateral inferior pubic rami—providing rigid and reliable fixation points not typically available in APR-related repair. For posterior fixation, which is technically more demanding, a cord-like fibrous structure was identified posterior to the hernia orifice on both preoperative digital palpation and intraoperative exploration. This structure was presumed to represent remnants of the transverse perineal muscle, perineal body, and perineal membrane, which have been described as fibromuscular structures in male anatomical studies.^8)^ Its preoperative identification allowed the surgical plan to include posterior fixation with confidence, and its robustness intraoperatively permitted tension-free mesh repair.

Beyond the selection of fixation points, the geometry of the mesh itself warrants careful consideration in this anatomical setting. The pelvic floor has a concave, bowl-shaped 3D anatomy, in contrast to the relatively flat anterior abdominal wall. Placement of a conventional circular or rectangular mesh in this confined space may result in mesh redundancy, causing wrinkling or folding. Mesh deformation adjacent to the bowel may, in particular, increase the risk of poor tissue integration or bowel-related complications. To improve conformability to the 3D pelvic floor contour while maintaining sufficient overlap, we tailored the mesh into a 4-armed configuration. Although this shape may theoretically reduce coverage in the diagonal directions, each arm was designed to provide approximately 3 cm of overlap beyond the hernia defect margin. The mesh was further reinforced with approximately 10 circumferential interrupted sutures along the rim of the hernia orifice. Moreover, unlike the anterior abdominal wall, the pelvic floor is not continuously subjected to strong lateral distracting forces. In this biomechanical setting, mesh conformability and stable multi-point fixation may be more important than achieving a perfectly planar mesh configuration. Taken together, the step-wise reconstructive strategy described here—preoperative identification of stable fixation points, geometry-adapted mesh tailoring, and biomechanically informed multi-point fixation—may offer a reproducible framework for surgeons encountering this rare complication, although longer follow-up will be needed to confirm the durability of this approach.

## CONCLUSIONS

We report a rare case of secondary anterior perineal hernia in a male patient following RARC with urethrectomy. To our knowledge, this is one of the first individually reported cases in a male patient in the English-language literature. A transperineal approach allowed safe and effective mesh repair using the posterior surface of the pubic bone, bilateral inferior pubic rami, and perineal fibrous remnants as stable fixation points. Surgeons should be aware of this rare but clinically significant complication following radical cystectomy and consider utilizing anatomically stable fixation points when planning reconstruction.

## DECLARATIONS

### Availability of data and materials

Data sharing is not applicable since no datasets were generated or analyzed during the present study.
